# Introducing Virtual Shared Medical Appointments as a Novel Treatment Platform for Functional Movement Disorders

**DOI:** 10.5334/tohm.921

**Published:** 2024-07-04

**Authors:** Saar Anis, Megan Zelinsky, Umar Shuaib, Emma Hartwig, Melissa Simpson, Hubert H. Fernandez, Taylor Rush, Xin Xin Yu

**Affiliations:** 1 Center for Neurological Restoration, Neurological Institute, Cleveland Clinic Foundation, Cleveland, Ohio, United States

**Keywords:** functional movement disorders, virtual shared medical appointments

## Abstract

The landscape of medical care has rapidly evolved with technological advancements, particularly through the widespread adoption of virtual appointments catalyzed by the COVID-19 pandemic. This shift has transcended geographical barriers, enhancing access for underserved populations and those with disabilities to specialized healthcare providers. A notable development stemming from this trend is the emergence of virtual shared medical appointments (VSMAs), which integrate group-based education with telemedicine technology. While VSMAs have demonstrated efficacy in conditions such as obesity, diabetes, and neurological disorders, their effectiveness in managing Functional Movement Disorders (FMD) is currently under investigation. FMDs pose unique challenges in diagnosis and acceptance, with high rates of misdiagnosis and treatment delays. VSMAs offer a promising solution by providing educational modules and fostering peer support among patients with similar diagnoses. At the Cleveland Clinic Center for Neurological Restoration, VSMAs have been embraced to enhance care standards for FMD patients. The program facilitates educational sessions and follow-up meetings to improve treatment adherence and psychological well-being. Early outcomes indicate increased patient acceptance and engagement, with significant program growth observed. Ongoing research aims to evaluate stakeholder perspectives and refine session content to further reduce stigma and the healthcare burden associated with FMDs.

In the rapidly evolving medical landscape, technological advancements have played a pivotal role in reshaping patient care paradigms. One of the most impactful shifts, precipitated by the COVID-19 pandemic, has been the widespread adoption of virtual appointments. While this innovation has certain drawbacks, its most notable advantage lies in its ability to transcend geographical barriers and enable patients from underserved areas or with disabilities to access consultations from specialized providers.

As an offshoot, another noteworthy development is the emergence of virtual shared medical appointments (VSMAs), representing an innovative fusion of traditional medical consultations with group-based education and telemedicine technology. VSMAs are an efficacious platform in various health conditions such as obesity, diabetes, and neurological disorders such as multiple sclerosis [1,2,3]. By bringing together multiple patients with similar health concerns in a single session, healthcare providers can address common issues, enhancing overall provider efficiency. Additionally, patients benefit from interacting with others facing similar health challenges, offering peer support.

Functional Movement Disorders (FMDs) pose unique challenges in both diagnosis and acceptance [4]. They affect up to 25% of patients who visit specialized movement disorder clinics [5,6,7]. Despite high prevalence, patients often face skepticism and misdiagnosis, leading to prolonged suffering and difficulties accessing appropriate care [8]. There is a scarcity of specialized centers equipped with multidisciplinary teams for FMDs treatment. Even upon receiving a diagnosis, there may remain barriers to acceptance and commitment to treatment [9]. Long-term follow-up studies reveal a poor prognosis with 50–90% of FMDs patients experiencing ongoing symptoms [10,11], many of whom become worse, especially when treatment begins later than 6–12 months from symptom onset [12]. This treatment gap disproportionately affects underserved populations, for whom timely and sustained intervention are difficult to obtain due to logistical and resource limitations. VSMAs present a promising solution to these challenges.

At a previous conference titled “Controversial Labels and Clinical Uncertainties: Psychogenic Disorders, Conversion Disorder, and Functional Symptoms,” in Atlanta, a roadmap was presented for reducing stigma and improving care for Functional Neurological Disorders (FNDs) [12]. It emphasized “empowering patients with FNDs to be heard and to drive changes in care” and “reducing isolation for clinicians by providing formal training and establishing multidisciplinary care teams and support networks”. A VSMA program can effectively address these goals and serve as a valuable learning opportunity for providers lacking multidisciplinary teams, with the ability to shadow specialized centers.

The Cleveland Clinic Center for Neurological Restoration, where the FMD program is housed, has enthusiastically embraced VSMAs to enhance care standards for individuals living with FMDs. The development of this treatment modality was supported by the Office of Patient Experience and training workshops offered by our institution. These workshops provided guidance on implementing and billing for VSMA, and best practices for workflow. Regular check-ins and webinars were also provided. Our three-year experience with VSMAs has underscored its profound benefits, offering hope for both patients and clinicians. Here we outline the structure of our VSMA program and highlight the notable advantages we have observed.

Once a diagnosis of FMD is confirmed, patients are extended an invitation to participate in VSMA sessions. Typically, each VSMA comprises a group of up to 10 patients. The initial VSMA session is dedicated to an educational module focused on providing patients and their loved ones with information to help them better grasp the diagnosis and rationale behind treatment approach. This session typically lasts for 90 minutes, primarily facilitated by a movement disorder specialist or an advanced practice provider. The inclusion of a nurse facilitator, who assists with logistical aspects, was introduced during implementation to address the technological access barriers experienced by some of our patients. The education module begins with a discussion of ground rules, going over expectations and emphasizing the importance of confidentiality and respectful participation. Each patient then introduces themselves, describing their current state of health and treatment, as well as their expectation from the visit to the extent they are comfortable. This is followed by an interactive presentation on general knowledge of FMDs including prevalence, phenotypes, diagnostic criteria, treatment structure, and overall treatment approach and its scientific rationale. Successful stories are shared with patient permission to instill hope and provide real-life examples of FMDs treatment journey. These elements aim to enhance therapy adherence and follow-up, alleviate psychological stress, and foster connections among patients who share a similar diagnosis. Finally, at the end of the session the facilitator makes sure that all patients have proper follow up with their treatment providers.

A subsequent milestone in the program’s development was the creation of follow-up sessions titled “Living Well with FMD”. These sessions continue to provide educational content on symptom management skills while fostering a supportive environment for patients to share their experiences and track their treatment progress. These sessions are accessible to all individuals interested in managing their FMD symptoms and are held monthly for 90 minutes. They cover a range of topics including goal setting, relaxation techniques, sleep hygiene, cognitive reframing strategies, imagery techniques (autogenics), distress tolerance, mindfulness, crisis management, relapse prevention, social support, and meditation, using the framework of cognitive behavioral therapy.

Thus far, our experience has shown promising outcomes, with patients reporting increased acceptance of their diagnosis and a greater willingness to engage in treatment following participation in the VSMA sessions. The program has gained traction with continuous growth of more than 300% from 2021 to the end of 2023 in terms of the number of patients and sessions conducted. Figure 1 illustrates our novel treatment framework and the increasing numbers of sessions and patients involved.

**Figure 1:**
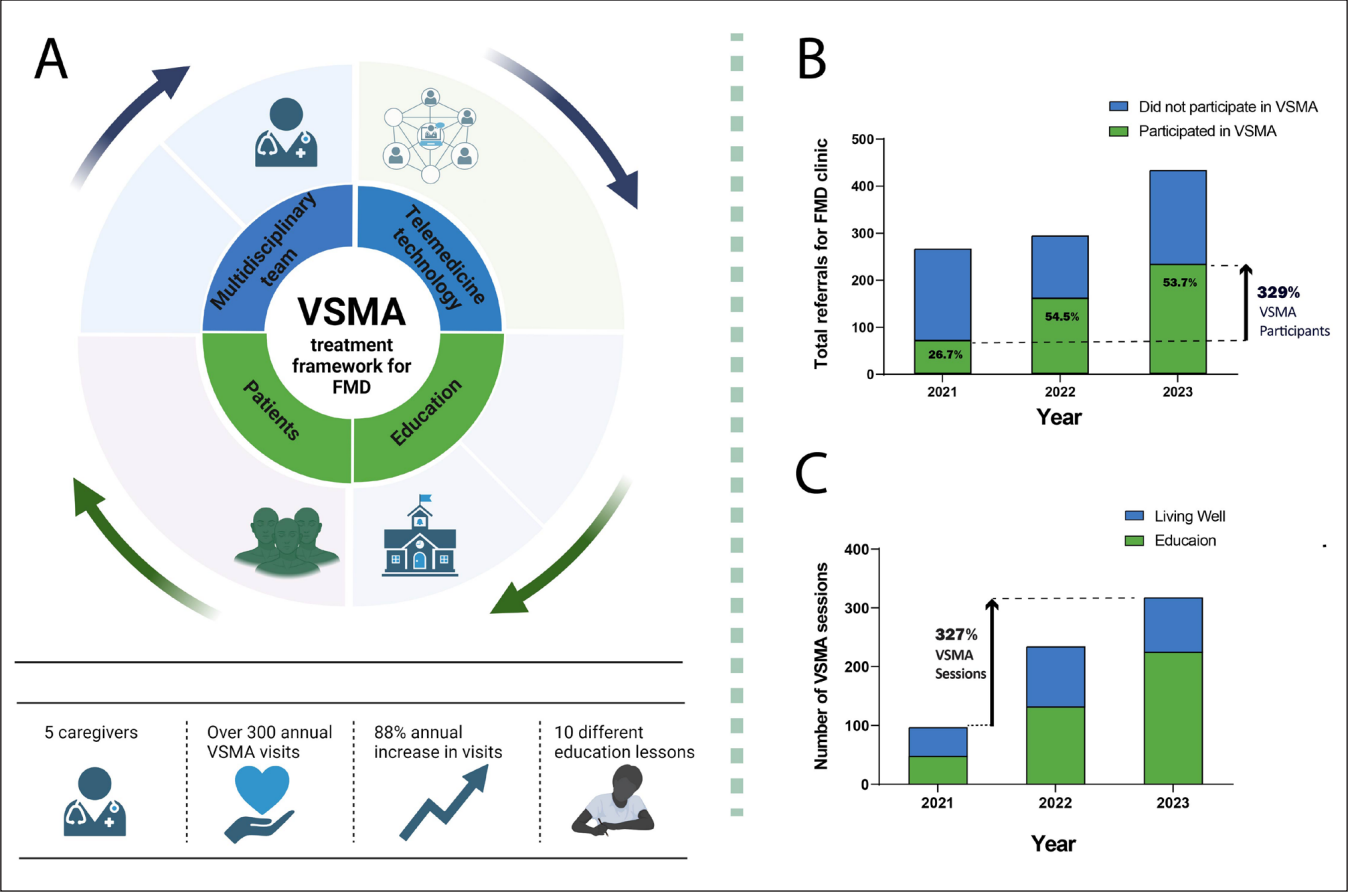
**A**, our proposed model; **B**, patients participating in VSMA relative to total referrals to FMD clinic; **C**, number of education and living well sessions out of all VSMA sessions.

Further research is essential to assess the impact from stakeholders’ perspectives, refine content, and understand how these meetings enhance diagnosis acceptance, combat stigma, and reduce healthcare burden. To address these objectives, a new 7-item questionnaire has been implemented to assess: patient satisfaction levels; relevance of content provided; confidence in the diagnosis; adherence to treatment; and willingness for future participation. Investigation into barriers including privacy concerns, session timing, group sharing reluctance, psychological factors, and technological challenges is crucial for effective implementation.
